# Better to Be Alone than in Bad Company: Cognate Synonyms Impair Word Learning

**DOI:** 10.3390/bs10080123

**Published:** 2020-07-29

**Authors:** Eneko Antón, Jon Andoni Duñabeitia

**Affiliations:** 1 Humanitate eta Hezkuntza Zientzien Fakultatea, Mondragon Unibertsitatea, 20500 Arrasate/Mondragón, Spain; eanton@mondragon.edu; 2 Centro de Ciencia Cognitiva (C3), Universidad Nebrija, 28015 Madrid, Spain; 3 Department of Languages and Culture, The Arctic University of Norway, 9019 Tromsø, Norway

**Keywords:** second language learning, word learning, cognate effect, synonymy, picture word association

## Abstract

The effects of cognate synonymy in L2 word learning are explored. Participants learned the names of well-known concrete concepts in a new fictional language following a picture-word association paradigm. Half of the concepts (set A) had two possible translations in the new language (i.e., both words were synonyms): one was a cognate in participants’ L1 and the other one was not. The other half of the concepts (set B) had only one possible translation in the new language, a non-cognate word. After learning the new words, participants’ memory was tested in a picture-word matching task and a translation recognition task. In line with previous findings, our results clearly indicate that cognates are much easier to learn, as we found that the cognate translation was remembered much better than both its non-cognate synonym and the non-cognate from set B. Our results also seem to suggest that non-cognates without cognate synonyms (set B) are better learned than non-cognates with cognate synonyms (set A). This suggests that, at early stages of L2 acquisition, learning a cognate would produce a poorer acquisition of its non-cognate synonym, as compared to a solely learned non-cognate. These results are discussed in the light of different theories and models of bilingual mental lexicon.

## 1. Introduction

Almost every individual has been exposed, at least to a certain degree, to a language other than the one she speaks. This is true now more than ever, considering that we live in a globalized world and that we are increasingly exposed to tourism, international media, and several other sources of linguistic diversity. In such a scenario, learning a new language is a challenge that many of us face. The motivations, realities, and modalities for second language learning are various, but irrespective of this, people tend to assume that some languages are easier to learn than others. This conclusion is often drawn simply by comparing the target language with the known one(s). Even though cross-linguistic similarity can arguably be one of the key factors that determine how easy it can be for a learner to acquire a new language, the comparison between two languages is multifactorial, as they can be catalogued from very closely related to very distant, depending on the criteria and the dimension we are focusing on when comparing them, see [1,2]. Intuitively, people normally tend to classify two languages as more similar or more distant based on how much their lexicons look alike, and consequently, equate similarity with ease, being an “easy” language if the word forms of the two languages are similar, and “hard” if they are not.

Attending to the lexicon, the most salient similarity that one can observe between two languages are the words known as cognates. Cognates are words from different languages that considerably overlap in meaning and form [3,4]. For example, the words “important” in English and “importante” in Spanish share all but one letter at the orthographic form level, and they have the exact same meaning, thus being close-to-perfect cognates. Therefore, one can think that, when an English native speaker learning Spanish encounters “importante” for the first time, she will understand and internalize it immediately without much effort. Thus, it is not surprising that cognates hold a special status in psycholinguistics and second language acquisition research, and therefore, have been extensively studied (for a review, see [5,6]).

Cognates have been found to be usually more easily processed in many experimental paradigms, both in production and in comprehension tasks. For example, either identical (e.g., piano-piano) or nearly-identical cognates (e.g., important-importante) are translated faster and with higher accuracy than non-cognates in translation tasks [7,8,9], as well as named faster and with fewer errors in naming tasks [10,11]. Furthermore, cognates are faster recognized than matched non-cognates in lexical decision and word identification tasks, especially in the second language (hereafter, L2, see [12,13,14,15,16]).

The cognate advantage that is found in both production and comprehension tasks has been typically explained by means of the structure and architecture of the bilingual mental lexicon. Following the non-selective account of lexical access in bilinguals, as defended by models like the Bilingual Interactive Activation model (BIA, [17], and its extensions, the BIA + model, see [18,19], and more recently, the BIA + s model by [20]), when a word is presented, its corresponding translation equivalent in the other language is activated too, especially when the two words share the orthographic structure [21]. Thus, when a cognate is encountered by a bilingual speaker, similar orthographic representations are activated in both languages, spreading activation to the common semantic representation, as it is widely accepted that the semantic system is shared between the two languages of a bilingual speaker [17,19,22,23,24,25,26,27]. This semantic activation, in turn, makes the reading, translation, learning, or recognition of the cognate word faster as compared to non-cognates via top-down modulation [19]. In production tasks, the process is even more straightforward. The initial activation at the semantic level of an element that is to be named automatically coactivates its corresponding translation equivalents, and in this way, cognate translations will activate similar or even the same phonological units in both languages, making it easier to utter cognates than non-cognates [28,29].

As a consequence of the above-mentioned facilitation effects and the stronger activation processes that cognates undergo, several studies have demonstrated that cognates are commonly faster retrieved from memory and less likely to be forgotten than non-cognates. Hence, cognates are easier to learn than non-cognates ([30,31]; for an overview, see [32,33]). Obviously, this has very important implications for any L2 learning scenario and any word learning method or paradigm in general. For example, the meaning of unknown cognate words can be successfully inferred by native speakers or learners after explicit instruction in word inferencing strategies [34], and relying on the L1 of English language learners has been shown to be a successful way to develop vocabulary knowledge in English, if both languages share cognates [35]. The role of the cognates is even more relevant in vocabulary learning at early stages of the language learning process since cognates have been found to be the guiding word types at the lowest levels of language competence [5]. Usually, vocabulary learning at these stages is deeply rooted in paired-associate learning, presenting the learners with pairings of their native-language words and their foreign-language translation equivalents (e.g., table-mesa for an English-Spanish combination). This way, an already known concept (i.e., a concept that the speaker can name in her mother tongue or L1) acquires a new lexical label in the L2 by means of its L1 counterpart (namely, an L1-mediation process; see [24]). Admittedly, the L1-L2 translation equivalent mapping is not the only instruction way, but it is still widely used at different levels of language teaching. Other methods usually involve picture-association methods, where the L2 word is presented together with a picture of the concept it represents [36,37], which has been shown to produce a superior recall of the learned L2 words as compared to a similar situation with L1-L2 word pairs [38]. Nonetheless, and irrespective of the teaching method employed, it has been repeatedly shown that cognates are easier to learn than non-cognates in adults [30,31,33] and children [39], even if the learners are not intentionally made aware of the cross-linguistic similarity [40].

As we become experts in the second language and move forward in the language learning process, we progressively encounter and have access to more words in that language. Once a new word is learned and stored in the bilingual mental lexicon, this item develops and strengthens connections within the system at semantic, lexical, and sub-lexical levels, both within and between languages [21,28,41]. Interestingly, the representations that already exist in the mental lexicon might determine the ease with which a new item can be learned and incorporated. For example, in a study exploring the effects of synonymy, Webb [42] discovered that during L2 word learning, words with already known synonyms (e.g., “locomotive”, whose high-frequency synonym “engine” was already known by participants) were easier to learn than words without a known synonym (e.g., “pawn”, “reef”, or “spear”). Considering that synonyms—words belonging to the same language that differ in form but highly overlap in meaning—have an impact on word learning, and that cognates—words belonging to different languages but that overlap in form and meaning—are easier to learn, in the current study, we asked whether cognate synonyms would have a differential effect in word learning as compared to other types of words. Concretely, we posed the question of whether L2 non-cognate words with a cognate synonym are learned differently from L2 non-cognate words with no synonyms at all. As a way of illustration, and imagining a Spanish native speaker learning English, we were interested in knowing if she would learn differently “couch” than “mirror”, considering that the first word has “sofa” as a synonym, which has a cognate translation equivalent in Spanish (“sofá”).

To this end, Primary and Secondary school students learned translations of Basque concrete nouns in a new fictional alien language. Concrete nouns were used as they are better learned than abstract nouns [36,43,44]. The translations were associated with visually presented pictures in order to boost the connections between the semantic system and the new lexical items [45]. Pseudo-words (i.e., words belonging to a fictional foreign language) rather than real words from a real foreign language were used to rule out any possible influence of pre-existing knowledge of the experimental materials. Participants learned different sets of cognate pseudo-words (similar to the word in participants’ L1, Basque) and non-cognate pseudo-words (with no connection to participants’ L1, Basque). Critically, some of the non-cognate and cognate pseudo-words were semantically related in pairs, as each one of the cognate pseudo-words had a synonym in the non-cognate set. In contrast, other non-cognate pseudo-words had no synonyms (see Materials and Procedure for further details). Thus, we had three kinds of pseudo-words: cognates, non-cognates with a cognate synonym, and non-cognates without a synonym. These would correspond to the words “sofa”, “couch” and “mirror” in the above-mentioned example. We had different predictions for each one of the types of items. First of all, we predicted that cognates would be learned better than the rest of the word types [18,39,46]. However, more importantly, we expected that the cognate facilitation effect would drag all the attention to the cognate synonym, harming the learning of its non-cognate synonym. In other words, the easiness of learning “sofa” would make it preferable for learning, and “couch” would become less salient and harder to learn. On the other hand, non-cognates without a cognate synonym would not suffer from this cognate attraction effect, and “mirror” would be learned better than “couch”, as “sofa” is dragging the attention from “couch” but nothing is prevailing over “mirror”.

## 2. Materials and Methods

### 2.1. Participants

In this study, 462 students from Olabide Ikastola school in Vitoria-Gasteiz (Basque Country, Spain) took part. The students ranged from 9 to 17 years of age (5th-year Primary school to 2nd-year Secondary school; 212 females; mean age = 12.99, SD = 2.25). They were immersed in a Basque education system, receiving formal schooling using Basque as a vehicular language.

### 2.2. Materials

A total of 40 Basque words and 60 pseudo-words were used in this experiment. The 60 pseudo-words were created to be the Basque words’ alien-language translations. First, a list with 40 common concrete Basque nouns was created and then divided into two sets of 20 words each (hereafter, sets A and B). There were no significant differences between the length, frequency, and orthographic neighborhoods of the words in sets A and B (all *p* > 0.4). Each of the 20 words in set A had two possible pseudo-word translations associated, one being a cognate (20 Cognate pseudo-words, C) and the other one being a non-cognate (20 Non-Cognate pseudo-words, NC). Consequently, the C and NC pseudo-words associated with each Basque word were synonyms (e.g., for the Basque word for bone, “hezur”, a cognate and a non-cognate pseudo-word were created, “hezor” and “iheba”, respectively). On the other hand, each of the 20 words in set B had only one non-cognate pseudo-word translation associated in the alien language (20 Unique Non-Cognate pseudo-words, UNC; e.g., the pseudo-word “tirka” for the Basque word for flower, “lore”).

The C set of pseudo-words was created by either adding a suffix at the end of the original Basque word (e.g., “arkatzoz” from the word “arkatz”, meaning pencil), by changing a letter of the original word (e.g., “sigar” from the word “sagar”, meaning apple), by removing a letter from the original word (e.g., “aulk” from the word “aulki”, meaning chair), or adding a letter to the original word (e.g., “zubiu” from the word “zubi”, meaning bridge). The pseudo-words in the NC and UNC sets were created by randomly combining legal Basque bigrams and trigrams. The two sets of non-cognates (NC and UNC) were not significantly different in length and in Levenshtein distance with respect to their original Basque translation words (all *p* > 0.2).

For the picture-association learning, two-dimensional drawings depicting each of the concepts named by the original 40 Basque real words were selected from the MultiPic Database [47]. The full list of words and pseudo-words can be seen in Appendix A and the drawings can be found at Supplementary https://doi.org/10.6084/m9.figshare.12582572.

### 2.3. Procedure

Participants completed the experiment during school hours at the school facilities, in the computer room. The experiment was conducted using LimeSurvey©, and participants used headphones to assure privacy. To avoid participants benefitting from comparing and contrasting their results, as well as to prevent exhaustion due to the memorization of the large number of words from the original list, 10 pseudo-randomized lists were created. Each list consisted of 20 real Basque words, 10 of which were paired with UNC pseudo-word translations, while the other 10 had both C and NC associated translations. Thus, each participant was presented with 50 items, 20 words and 30 pseudo-words (10 C, 10 NC, and 10 UNC) in total.

The experiment was conducted as follows: Firstly, participants were randomly assigned to one of the 10 experimental lists. Then, they were presented with a cartoon picture of a friendly alien named Klorg, and they were told that he was from a faraway planet who came to Earth to teach them his language. Then, the exposition phase started, following a picture-association paradigm where participants were presented with a drawing depicting one of the original 20 Basque words. If the concept had one only possible translation in the alien language (namely, it was one of the 10 concepts from the UNC list), the alien would say an invented sentence in which the critical pseudo-word was included twice:

- “Iski nual gruain **tirka**, fronum gro glu **tirka**”.
  (“What you see here is called **tirka**, we call it **tirka**”).

If the picture represented a concept with two possible translations in the alien language (namely, it was one of the 10 concepts from the C and NC list), the alien would say a sentence very much like the one from the UNC set, but on this occasion, each pseudo-word was mentioned only once:

- “Iski nual gruain **hezor**, fronum gru ansi **iheba**”.
  (“What you see here is called **hezor**, and we also call it **iheba**”).

**Figure 1:**
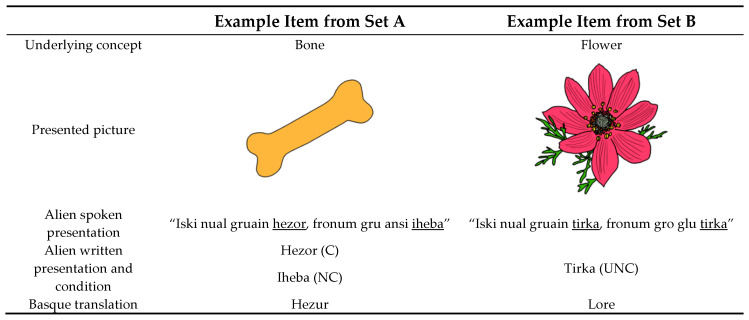
Schematic representation of the presentation of the materials and the experimental conditions.

The sentences were recorded by a native Basque speaker. Together with the recordings, the target pseudo-words were presented on the screen in a written form. If the target concept had two possible translations (NC and C), one pseudo-word was presented above the drawing in capitalized Helvetica font, size 20 pt., and the other one right below it in lowercase Georgia font, size 20 pt. If the target concept had a unique translation (UNC), the pseudo-words were presented twice on the screen in the same styles as described above. Participants were exposed to each token once (see Figure 1 for a schematic representation of the materials).

After the exposure or learning phase, two recognition tests were carried out. The first recognition test consisted of a picture-word matching task in which participants were presented with a drawing from the learned set on the screen, and they were instructed to select the alien word corresponding to the depicted concept. They were reminded that some concepts could have only one translation, and others, two. Participants were presented with one drawing at a time at the top of the screen, and with a list of all the 30 alien words (namely, the UNC, C and NC pseudo-words) that they were exposed to. They had to choose the string or strings describing said concept (they could select more than one item, given that some pictures were associated with two pseudo-words in the exposure phase). Participants had no time limit to respond to each item.

The second recognition task consisted of a translation recognition paradigm in which they were asked to help Klorg, the alien. Klorg would tell them a word in his language, and they had to select the Basque translation of it. Participants would see the alien word at the top of the screen, and below, they had the list of the 20 Basque words that described the concepts they had learned. They had to choose the correct translation with no time limit. Only the non-cognate pseudo-words (i.e., UNC and NC conditions) were presented, given that presenting them with the cognate words (i.e., C condition) would have been too easy and not too informative, since responses could have been driven by the obvious visual similarity (e.g., “hezor”-“hezur”). Thus, in this task, there was only one correct answer to each trial.

## 3. Results

Accuracy was collected in both recognition tasks, and results were analyzed separately for the picture-word matching task and for the translation recognition task (see Table 1 for the descriptive statistics).

First, in the picture-word matching task, the percentages of correct recognition of items belonging to set A and B were compared. For set A, a response was considered correct if the participant chose the correct C pseudo-word, the correct NC pseudo-word, or both (see the Total score in Table 1). Set B only had one possible correct answer. A paired samples t-test analysis indicated that items belonging to set A were correctly recognized significantly more often than those belonging to set B (i.e., more than twice as much; *t* (461) = 34.9, *p* < 0.01, *d* = 1,62; see Table 1). In order to explore the differences between the specific types of correct responses in set A (choosing only C, choosing only NC, or choosing both C and NC), a one-factor ANOVA with Type of Choice as a single factor with three levels (C only|NC only|C and NC) was conducted. Results indicated a significant effect (*F* (2,922) = 509, *p* < 0.01), and planned comparisons using Bonferroni-corrected t-tests indicated that responses correctly choosing only the cognate pseudo-words (C only) happened more often than responses correctly choosing only the non-cognate pseudo-words (NC only) (*t* (922) = 30.09, *p* < 0.01), and than responses choosing both cognate and non-cognate pseudo-words (C and NC) (*t* (922) = 24.23, *p* < 0.01). This showed a clear-cut cognate facilitation effect. Responses choosing correctly both the C and the NC pseudo-words (C and NC) also happened significantly more frequently than responses correctly choosing only the NC pseudo-words (NC only) (*t* (922) = 5.86, *p* < 0.01).

The analysis carried out on the accuracy in the translation recognition task in which participants had to select the correct Basque translations for each of the alien language words showed that UNC translations belonging to set B were recognized better than NC translations belonging to set A (*t* (461) = 9.30, *p* < 0.01, *d* = 0.43) (see Table 1).

## 4. Discussion

The results presented in this article strongly suggest that cognates, their non-cognate synonyms, and non-cognates with no synonyms are learned differently. First of all, we observed a cognate superiority effect in word learning. In the picture-word matching task (namely, the first recognition task), participants correctly recognized the concepts associated with cognate names (set A) more than twice as often as the ones with only non-cognate names (set B). As predicted in the Introduction, cognate items hold a special status in the lexicon by which they are easier to process, integrate, and later recognize. As proposed by Nation [48], the “learning burden” of cognate words would be very light, as they heavily rely on participants’ previous knowledge of their L1. This finding is consistent with the literature showing that cognate words are better and faster learned than non-cognates, both in adult and children populations, most probably due to the semantic co-activation caused by highly similar lexico-orthographic representations [18,39,46]. As an indicator of the strength of the cognate facilitation effect, it is worth noting that in set A, the concepts were presented with their C and NC translations, and consequently, the cognate pseudo-words were presented on the screen half as often as the items from set B, where the non-cognate pseudo-words were presented twice for each item. Still, concepts from set A were correctly remembered more than twice as often as those from set B.

In line with current findings, it should be considered that cognates seem to attract all the attention during the learning process, over and above their possible non-cognate synonyms. This becomes clear after a further inspection of the specific choices made by the participants in set A. The higher percentage of correct response for set A was clearly driven by the cognate translations. As shown in Table 1, correct responses associated exclusively with a cognate choice (i.e., responses correctly identifying only cognates) occurred much more often than correct responses that included non-cognates (i.e., correct responses identifying only the non-cognate pseudo-word and correct responses identifying both the non-cognate and the cognate pseudo-word). Hence, in the presence of a cognate together with an alternative lexical tag for a given element that is a non-cognate, only the former serves as an attractor, facilitating its learning, on the one hand, and harming the learning of the non-cognate counterpart, on the other. The potential negative impact that a cognate synonym might have in word learning for other competing lexical forms should not be overlooked. Not only were the non-cognates from set A (NC) learned much worse than their cognate synonyms (C), but they were also remembered less than the non-cognate pseudo-words from set B (UNC), and this occurred both in the picture-word matching task and in the translation recognition task.

The difference between NC and UNC items could be due to a frequency effect, as participants saw the UNC pseudo-words twice on the screen in each presentation, while the NC pseudo-words were presented once (and together with C). However, the superior recall of non-cognates without synonyms as compared to non-cognates with synonyms could also be explained by the attention attraction produced by the cognates. As learning a cognate pseudo-word would suffice to name and recognize the concept presented in set A, the importance of the non-cognate synonym decreases. In set B, in the absence of cognates, the non-cognates are the only elements with which the concept could be named and recognized. Indeed, when synonym pairs have to be learned, attention and effort needed to learn them seem to be higher, as several studies have shown that acquiring pairs of synonyms is harder than acquiring pairs of unrelated words [49], indicating that the words with closer semantic relationship are harder to learn than unrelated sets (see also [50,51,52]). That might have been the case in our study as well. Learners of a language usually learn words that allow them to convey new information early in the process of learning a language, and they learn synonyms later in said process [42], and they seem to learn and use non-cognates later in the process, too [5]. As novice learners of a new language, our participants successfully learned the new lexical labels for the concepts, but in the case of multiple options for the same concept (namely, in the presence of synonyms), they prioritized the less effortful option—the cognates.

Summarizing, our results clearly indicate that cognates are learned more easily than non-cognates during early stages of L2 learning, even when they are presented in synonym pairs. Our data also suggest that, at early stages of L2 learning, non-cognates without a synonym could be better learned than non-cognates with a cognate synonym. In practical terms, this means that when a Spanish speaker is learning English and she encounters the words “couch”, “sofa” and “mirror” for the first time, she would learn them at different paces. She will rapidly acquire “sofa” given that it almost completely overlaps with its translation “sofá”. However, and as suggested by the current results, she will learn “mirror” easier than “couch”. The cognate status of “sofa” seems to make “couch” harder to learn, or less important or necessary for the system. From a communicative perspective, why would a person who is starting to learn a language spend time, attention, and energy in learning another lexical item (“couch”) for a concept that she can already name using a word very similar to that of her native language (“sofa”)? She would rather invest in learning labels for concepts she cannot name yet (“mirror”).

Taking our data and previous findings together, it seems reasonable to conclude that the bilingual lexicon has a structure that initially favors the association of one single lexical item from the L2 to each known L1 word at the beginning of a learning process, favoring the learning of cognate items (given their overlap with the L1 counterparts) and disfavoring synonym learning (given that this would imply learning two new items that, in part, provide redundant information). Nonetheless, this is expected to change as a function of increased knowledge, proficiency, or use of the L2. Indeed, many models take into account the effect of proficiency in the L2 (like the BIA-d model, [53]; or the Revised Hierarchical Model, [24]) and its effects in the structure and connectivity between the different nodes of a bilingual mental lexicon. At higher proficiency levels, learners would probably be able to acquire more than one lexical label at a time for the same semantic representations (namely, synonyms). This prediction is reinforced by the fact that, at higher levels of proficiency, learners of a language rely much less upon cross-linguistic similarity as their L2 items are much less connected to their L1 equivalents than at lower levels (see [24,53,54]). Hence, L2 learners rely much less on their L1 as proficiency increases, and there is evidence showing an inverse relationship between the degree of reliance on cross-linguistic similarity and reading comprehension at intermediate L2 proficiency levels [5]. In fact, better L2 reading comprehension is achieved by novice learners that rely more on cross-linguistic similarity, but at intermediate or higher levels the effect reverses, and better L2 reading comprehension is achieved by learners who rely less on cross-linguistic overlap. Thus, further research would be needed in order to explore the way in which the acquisition of different lexical items (e.g., cognate and non-cognate synonyms) is carried out at different stages of proficiency in second language learning.

## 5. Conclusions

Summarizing the results, in the present study, we presented evidence of a cognate superiority effect in L2 word learning, as well as a negative impact of having a cognate synonym in L2 word learning. This piece of work adds relevant information for second language learning, and it opens future lines of research and posits new questions. The question of whether proficiency or language use and exposure can have an impact on the capacity to learn the different kinds of words presented here, should be explored in future studies to plan teaching strategies accordingly.

## Figures and Tables

**Table 1:** Mean percentages of correct responses given to each condition in both recognition tasks. Standard deviation values are displayed in the second row for each condition in each task.

	Picture-Word Matching					Translation Recognition	
	SET A				SET B	SET A	SET B
	C only	NC only	C and NC	Total	Total	NC	UNC
Mean	53%	6%	16%	76%	37%	20%	28%
Standard deviation	25%	10%	25%	24%	29%	24%	28%

## Supplementary Materials

The drawings used in the experiment are available at https://doi.org/10.6084/m9.figshare.12582572.

## Appendix A

**Table A1:** Pseudo-words used in the experiment, their Basque and English translations, and the pictures used in the picture-word association task.

SET A					SET B			
Concept	Basque Word	Associated Picture	C	NC	Concept	Basque Word	Associated Picture	UNC
bird	txori	PICTURE_430	txor	redal	hair	ile	PICTURE_102	artune
dog	txakur	PICTURE_707	txakuroz	krogo	table	mahai	PICTURE_110	flobor
root	sustrai	PICTURE_245	sustraioz	muno	beard	bizar	PICTURE_159	tipu
pencil	arkatz	PICTURE_654	arkatzoz	glapa	head	buru	PICTURE_182	edupe
river	ibai	PICTURE_86	ibaiu	fluti	wheel	gurpil	PICTURE_2	lula
blackboard	arbel	PICTURE_402	arbelu	bleti	stone	harri	PICTURE_279	plaglo
bone	hezur	PICTURE_24	hezor	iheba	flower	lore	PICTURE_312	tirka
apple	sagar	PICTURE_552	sigar	tadru	fish	arrain	PICTURE_324	gribe
lip	ezpain	PICTURE_513	ezpainoz	furron	coin	txanpon	PICTURE_383	bluna
key	giltza	PICTURE_78	giltzu	astes	snow	elur	PICTURE_390	baru
branch	adar	PICTURE_536	adaru	udigo	teacher	maisu	PICTURE_434	debrati
walnut	intxaur	PICTURE_119	intxauroz	burtiko	box	kutxa	PICTURE_508	truplo
finger	behatz	PICTURE_408	behat	kamut	cloud	hodei	PICTURE_599	plobo
bed	ohe	PICTURE_268	oheu	troka	honey	ezti	PICTURE_610	tupuk
cheese	gazta	PICTURE_72	gazt	oilop	barrel	kupel	PICTURE_630	gasi
bridge	zubi	PICTURE_178	zubiu	serta	thief	lapur	PICTURE_631	ripeda
hammer	mailu	PICTURE_216	meilu	betome	bee	erle	PICTURE_672	ibima
house	etxe	PICTURE_522	etxa	gufal	trousers	galtza	PICTURE_718	aban
chair	aulki	PICTURE_122	aulk	freba	heart	bihotz	PICTURE_83	gafo
leaf	hosto	PICTURE_318	host	tlobo	milk	esne	PICTURE_96	utepa
